# Propensity score matching analysis of cisplatin-based concurrent chemotherapy in low risk nasopharyngeal carcinoma in the intensity-modulated radiotherapy era

**DOI:** 10.18632/oncotarget.5806

**Published:** 2015-10-19

**Authors:** Lu-Ning Zhang, Yuan-Hong Gao, Xiao-Wen Lan, Jie Tang, Zhen Su, Jun Ma, Wuguo Deng, Pu-Yun OuYang, Fang-Yun Xie

**Affiliations:** ^1^ Department of Radiation Oncology, Sun Yat-sen University Cancer Center, State Key Laboratory of Oncology in South China, Collaborative Innovation Center for Cancer Medicine, Guangzhou, Guangdong, China; ^2^ Department of Experimental Research, Sun Yat-sen University Cancer Center, State Key Laboratory of Oncology in South China, Collaborative Innovation Center for Cancer Medicine, Guangzhou, Guangdong, China

**Keywords:** concurrent chemotherapy, intensity-modulated radiotherapy, nasopharyngeal carcinoma, propensity score matching, survival

## Abstract

**Background:**

Patients with stage II nasopharyngeal carcinoma were reported to benefit from adding cisplatin-based concurrent chemotherapy to two-dimensional conventional radiotherapy. But this benefit becomes uncertain in the intensity-modulated radiotherapy (IMRT) era, owing to its significant advantage.

**Methods:**

We enrolled 661 low risk (T1N1M0, T2N0-1M0 or T3N0M0, the 2010 UICC/AJCC staging system) patients who underwent IMRT with or without concurrent chemotherapy. Particularly, patients with IMRT alone or IMRT plus cisplatin-based concurrent chemotherapy were equally matched using propensity-score matching method. Overall survival (OS), distant metastasis-free survival (DMFS) and locoregional relapse-free survival (LRFS) were assessed with Kaplan-Meier method, log-rank test and Cox regression.

**Results:**

Among 661 patients, IMRT alone achieved parallel OS (*P* = 0.379), DMFS (*P* = 0.169) and LRFS (*P* = 0.849) to IMRT plus concurrent chemotherapy. In the propensity-matched cohort of 482 patients, similar survival were observed between both arms (4-years OS 97.4% vs 96.1%, *P* = 0.134; DMFS 96.5% vs 95.1%, *P* = 0.763; LRFS 93.8% vs 91.5%, *P* = 0.715). In multivariate analysis, cisplatin-based concurrent chemotherapy did not lower the risk of death, distant metastasis or locoregional relapse. And this association remained unchanged in subgroups by age, sex, histology and stage.

**Conclusions:**

In this study, low risk nasopharyngeal carcinoma patients who underwent IMRT could not benefit from cisplatin-based concurrent chemotherapy.

## INTRODUCTION

Nasopharyngeal carcinoma (NPC) is a malignancy relatively rare in Europe and the United States [1] but highly endemic in Southern China [2] and Hong Kong [3]. Radiotherapy is the mainly standard treatment. A recent phase III randomized trial showed considerable survival benefit from the combined treatment of cisplatin-based concurrent chemotherapy and two-dimensional conventional radiotherapy (2DCRT) for patients with stage II (the Chinese 1992 staging system) of this disease [4]. However, since intensity-modulated radiotherapy (IMRT) was known to be superior to 2DCRT in local control [5], it is a pivotal question whether patients in low risk of relapse, distant metastasis or death [e.g. T1N1M0, T2N0-1M0 or T3N0M0, based on the 2010 International Union against Cancer/American Joint Committee on Cancer (UICC/AJCC) staging system] can still obtain significant benefit from the additional concurrent chemotherapy in the IMRT era. Unfortunately, there is no convincing evidence from any large scale completed randomized controlled trial, due to the low incidence of NPC in most area, the small proportion of patients with early stage, and the recent application of IMRT in the endemic area. To address this question, we retrospectively analyzed data of 661 patients with stage T1N1M0, T2N0-1M0 or T3N0M0 who received IMRT with or without concurrent chemotherapy. We especially compared the survival outcomes of IMRT alone with IMRT plus cisplatin-based concurrent chemotherapy in a propensity score matched cohort, which was likely to mimic randomized trials [6]. This shall provide valuable support for treatment guidelines and suggestion for the future randomized controlled trials.

## RESULTS

### Patients

A total of 661 patients were entered into this study. Initially, 254 (38.4%) and 407 (61.6%) patients were treated with IMRT alone and IMRT plus concurrent chemotherapy, respectively. Following propensity score matching, 241 patients treated with IMRT alone and 241 patients treated with IMRT plus cisplatin-based concurrent chemotherapy remained in the analysis. The matched patients in both arms had balanced characteristics (Table 1). The average dose of cisplatin delivered in the propensity-matched cohort was about 175 mg/m^2^.

**Table 1:** Baseline characteristics of nasopharyngeal carcinoma patients treated with intensity-modulated radiotherapy with or without concurrent chemotherapy

	The original unmatched cohort						The propensity-matched cohort					
	IMRT alone (*N* = 254)		IMRT+CC (*N* = 407)		*P*	Standardized difference	IMRT alone (*N* = 241)		IMRT+CC (*N* = 241)		*P*	Standardized difference
	No.	%	No.	%			No.	%	No.	%		
**Age**					**0.021**	**0.181**					**0.894**	**0.012**
Mean	48.31		46.24				47.99		47.85			
SD	12.30		10.49				12.22		10.31			
Median	46.50		45.00				46.00		47.00			
**Sex**					**0.540**	**0.049**					**0.917**	**0.009**
Male	189	74.4	294	72.2			179	74.3	180	74.7		
Female	65	25.6	113	27.8			62	25.7	61	25.3		
Histology*					**0.501**	**0.055**					**0.611**	**0.046**
II	8	3.1	17	4.2			7	2.9	9	3.7		
III	246	96.9	390	95.8			234	97.1	232	96.3		
**VCA-IgA**^†^					**0.160**						**0.628**	
<80	69	27.2	89	21.9		**0.123**	61	25.3	59	24.5		**0.019**
80–320	94	37.0	145	35.6		**0.029**	90	37.3	82	34.0		**0.069**
≥320	91	35.8	173	42.5		**0.137**	90	37.3	100	41.5		**0.085**
**EA-IgA**^†^					**0.107**						**0.592**	
<10	111	43.7	147	36.1		**0.155**	102	42.3	97	40.2		**0.042**
10–40	80	31.5	134	32.9		**0.031**	78	32.4	73	30.3		**0.045**
≥40	63	24.8	126	31.0		**0.138**	61	25.3	71	29.5		**0.093**
**T-stage**					**< 0.001**						**0.701**	
T1	74	29.1	96	23.6		**0.126**	73	30.3	69	28.6		**0.036**
T2	140	55.1	186	45.7		**0.189**	128	53.1	125	51.9		**0.025**
T3	40	15.7	125	30.7		**0.360**	40	16.6	47	19.5		**0.076**
**N-stage**					**0.503**	**0.053**					**0.296**	**0.095**
N0	104	40.9	156	38.3			92	38.2	81	33.6		
N1	150	59.1	251	61.7			149	61.8	160	66.4		
**Clinical stage**					**< 0.001**	**0.360**					**0.407**	**0.075**
II	214	84.3	282	69.3			201	83.4	194	80.5		
III	40	15.7	125	30.7			40	16.6	47	19.5		

Abbreviations: IMRT = intensity-modulated radiotherapy, CC = concurrent chemotherapy, SD = standard deviation, VCA = viral capsid antigen, EA = early antigen, IgA = immunoglobulin A

* Based on the criteria of WHO histological type (1991): II - Differentiated non-keratinising carcinoma, III - Undifferentiated non-keratinising carcinoma

† In accordance with the criteria adopted in previous studies

### Survival outcomes

**Figure 1:**
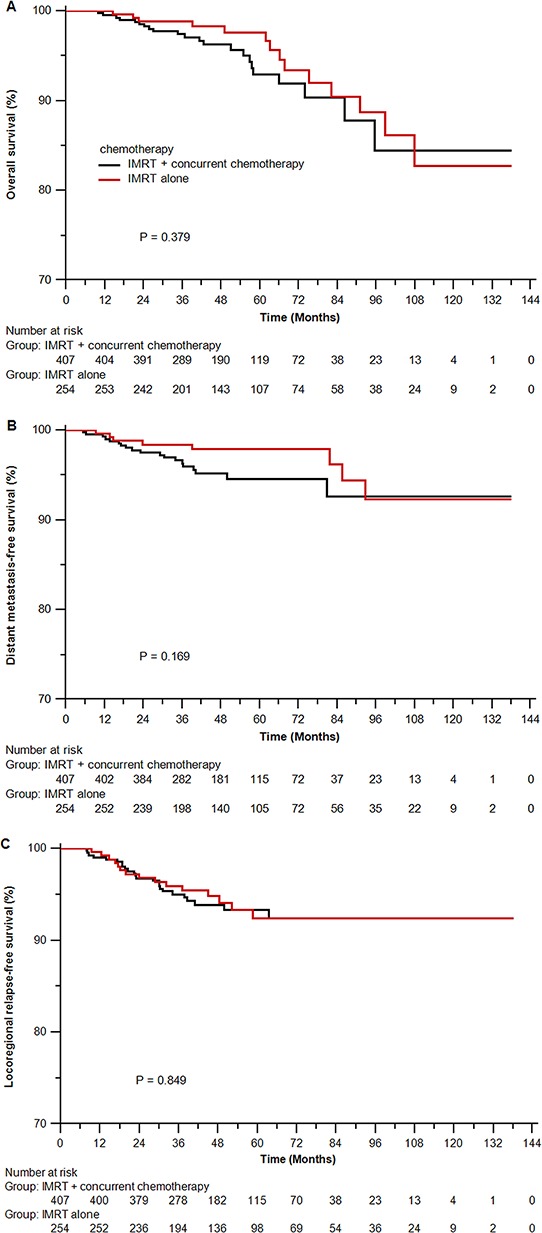
Kaplan-Meier survival curves for the IMRT alone arm and the IMRT plus concurrent chemotherapy arm in the original unmatched cohort of 661 patients **A.** overall survival; **B.** distant metastasis-free survival; **C.** locoregional relapse-free survival. IMRT = intensity-modulated radiotherapy.

In the original unmatched cohort of 661 patients, the median follow-up time was 51.2 months (10.9–138.0 months) for the IMRT alone arm and 46.7 months (10.0–138.0 months) for the IMRT plus concurrent chemotherapy arm, respectively. Overall, 4-years overall survival (OS), distant metastasis-free survival (DMFS) and locoregional relapse-free survival (LRFS) rates did not differ significantly between the two arms (OS 97.5% vs 95.8%, *P* = 0.379; DMFS 97.3% vs 94.8%, *P* = 0.169; and LRFS 94.1% vs 93.4%, *P* = 0.849; Figure 1A–1C). Accounting for age (continuous), sex, titers of immunoglobulin A against viral capsid antigen (VCA-IgA, < 80/80–320/≥ 320) and early antigen (EA-IgA, < 10/10–40/≥ 40), T-stage and N-stage in multivariate analysis, IMRT alone was not associated with higher risk of death, locoregional relapse or distant metastasis than IMRT plus concurrent chemotherapy. (Table 2).

**Table 2:** Summary of important prognostic factors in multivariate analysis

	The original unmatched cohort		The propensity-matched cohort	
	Hazard ratio (95% CI)	*P* ^†^	Hazard ratio (95% CI)	*P* ^‡^
**Overall survival**				
IMRT alone versus IMRT+CC	0.64 (0.31–1.31)	0.224	0.70 (0.34–1.44)	0.328
Age (continuous)	1.06 (1.03–1.09)	< 0.001	1.05 (1.011.10)	0.011
Sex	0.92 (0.43–1.99)	0.833	0.82 (0.26–2.55)	0.731
Histology	0.53 (0.16–1.80)	0.310	0.92 (0.10–8.03)	0.936
VCA-IgA	0.83 (0.45–1.53)	0.554	0.54 (0.27–1.07)	0.076
EA-IgA	1.20 (0.66–2.19)	0.554	1.77 (0.91–3.43)	0.091
T-stage	1.10 (0.53–2.28)	0.789	1.14 (0.48–2.70)	0.773
N-stage	1.06 (0.40–2.78)	0.913	0.72 (0.28–1.86)	0.499
**Distant metastasis-free survival**				
IMRT alone versus IMRT+CC	0.60 (0.26–1.42)	0.248	0.69 (0.30–1.58)	0.383
Age (continuous)	1.01 (0.97–1.04)	0.677	1.04 (0.99–1.10)	0.127
Sex	0.43 (0.15–1.26)	0.125	0.53 (0.15–1.91)	0.333
Histology	0.46 (0.11–1.95)	0.290	-	-
VCA-IgA	0.70 (0.34–1.46)	0.341	0.41 (0.18–0.92)	0.032
EA-IgA	1.72 (0.87–3.43)	0.122	2.08 (0.98–4.45)	0.058
T-stage	1.14 (0.50–2.62)	0.496	1.11 (0.37–3.32)	0.858
N-stage	2.17 (0.65–7.27)	0.210	1.36 (0.35–5.30)	0.661
**Locoregional relapse-free survival**				
IMRT alone versus IMRT+CC	1.01 (0.52–1.97)	0.974	0.82 (0.40–1.67)	0.586
Age (continuous)	1.01 (0.98–1.04)	0.617	1.00 (0.97–1.04)	0.941
Sex	1.72 (0.90–3.30)	0.099	1.67 (0.82–3.38)	0.158
Histology	-	-	-	-
VCA-IgA	1.13 (0.62–2.03)	0.695	0.99 (0.52–1.88)	0.980
EA-IgA	0.82 (0.46–1.46)	0.505	0.89 (0.46–1.71)	0.724
T-stage	1.57 (0.78–3.16)	0.207	1.74 (0.84–3.58)	0.133
N-stage	2.35 (0.87–6.39)	0.093	1.89 (0.69–5.23)	0.218

Abbreviations: CI = confidence interval, CC = concurrent chemotherapy, IMRT = intensity-modulated radiotherapy, VCA = viral capsid antigen, EA = early antigen, IgA = immunoglobulin A

† Adjusted for age (continuous), sex, histology, VCA-IgA (<80/80–320/≥ 320), EA-IgA (<10/10–40/≥ 40), T-stage and N-stage.

‡ Adjusted for the same covariates with a robust variance estimator to account for the clustering within matched pair.

**Figure 2:**
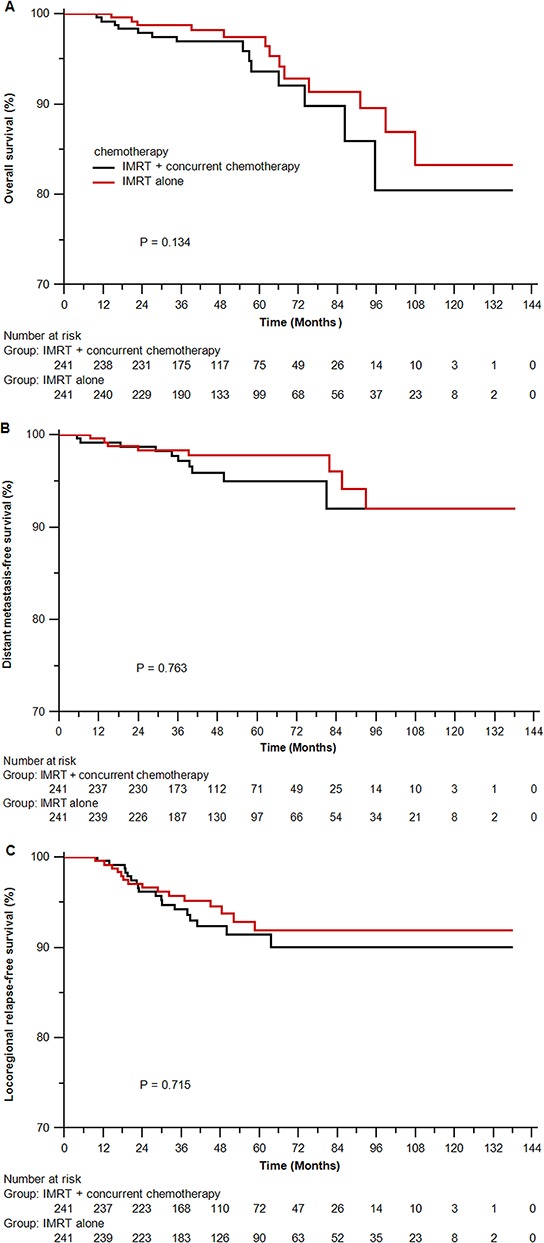
Kaplan-Meier survival curves for the IMRT alone arm and the IMRT plus cisplatin-based concurrent chemotherapy arm in the propensity-matched cohort of 482 patients **A.** overall survival; **B.** distant metastasis-free survival; **C.** locoregional relapse-free survival. IMRT = intensity-modulated radiotherapy. *P* values were calculated using stratified log-rank test by matched pairs.

In the propensity-matched cohort of 482 patients, the median follow-up time was 50.7 months (10.9–138.0 months) for the IMRT alone arm and 47.6 months (10.0–138.0 months) for the IMRT plus cisplatin-based concurrent chemotherapy arm, respectively. In univariate analysis, IMRT alone resulted in parallel survival to IMRT plus cisplatin-based concurrent chemotherapy (OS rates at 4-years 97.4% vs 96.1%, *P* = 0.134; DMFS rates at 4 years 96.5% vs 95.1%, *P* = 0.763; and LRFS rates at 4 years 93.8% vs 91.5%, *P* = 0.715; Figure 2A–2C). In multivariate analysis, IMRT alone was also highly comparable to IMRT plus cisplatin-based concurrent chemotherapy in risk of death, locoregional relapse and distant metastasis. (Table 2).

In subgroup analysis by age (<45/≥ 45 years), sex and histology in the propensity-matched cohort, IMRT alone showed no significant survival differences from IMRT plus cisplatin-based concurrent chemotherapy. In separate subgroup of stage T1N1M0, T2N0M0 and T2N1M0 (stage II), IMRT alone led to similar survival to IMRT plus cisplatin-based concurrent chemotherapy, independent of other covariates. With restriction to patients with stage T3N0M0 (stage III), the effect of cisplatin-based concurrent chemotherapy on OS, DMFS and LRFS was also independently insignificant. (Table 3).

**Table 3:** Subgroup analysis by prognostic factors in multivariate analysis in the propensity-matched cohort *

	Overall survival		Distant metastasis-free survival		Locoregional relapse-free survival	
	Hazard ratio (95% CI)	*P*	Hazard ratio (95% CI)	*P*	Hazard ratio (95% CI)	*P*
**Age**						
<45 ys	0.38 (0.08–1.89)	0.237	0.45 (0.09–2.19)	0.324	0.93 (0.34–2.57)	0.885
≥45 ys	0.87 (0.37–2.06)	0.752	0.87 (0.31–2.41)	0.789	0.73 (0.27–1.99)	0.539
**Sex**						
Male	0.56 (0.23–1.38)	0.209	0.78 (0.31–1.93)	0.589	0.74 (0.30–1.87)	0.526
Female	1.69 (0.35–8.04)	0.511	0.14 (0.01–1.31)	0.085	1.10 (0.33–3.65)	0.873
**Histology**						
II	-	-	-	-	-	-
III	0.77 (0.37–1.59)	0.473	0.69 (0.30–1.58)	0.383	0.82 (0.40–1.67)	0.586
**Stage**						
T1N1	0.58 (0.10–3.17)	0.527	0.35 (0.07–1.80)	0.209	0.94 (0.22–3.95)	0.930
T2N0	0.22 (0.02–3.11)	0.263	-	0.927	1.11 (0.07–18.44)	0.942
T2N1	1.01 (0.23–4.39)	0.993	0.47 (0.11–2.09)	0.325	1.12 (0.40–3.17)	0.827
T3N0	0.70 (0.11–4.57)	0.713	2.61 (0.20–33.48)	0.461	0.41 (0.07–2.50)	0.331

Abbreviations: CI = confidence interval

* Adjusted for age (continuous), sex, histology, VCA-IgA (<80/80–320/≥ 320), EA-IgA (<10/10–40/≥ 40), T-stage and N-stage with a robust variance estimator to account for the clustering within matched pair.

### Hematological toxicities

In the propensity-matched cohort, cisplatin-based concurrent chemotherapy significantly increased the incidence of grade 1–2 leucopenia, neutropenia, anemia and thrombocytopenia, and grade 3–4 leucopenia and neutropenia. (Table 4)

**Table 4:** Hematological toxicities in the propensity-matched cohort

	IMRT alone (*N*=241)	IMRT+CC (*N*=241)	*P*	Standardized difference
**Leucopenia**			**<0.001**	
Grade 1–2	87 (36.1%)	152 (63.1%)		**0.560**
Grade 3–4	7 (2.9%)	21 (8.7%)		**0.250**
**Neutropenia**			**<0.001**	
Grade 1–2	16 (6.6%)	92 (38.2%)		**0.817**
Grade 3–4	7 (2.9%)	12 (5.0%)		**0.107**
**Anemia**			**< 0.001**	
Grade 1–2	54 (22.4%)	153 (63.5%)		**0.912**
Grade 3–4	3 (1.2%)	4 (1.7%)		**0.035**
**Thrombocytopenia**			**<0.001**	
Grade 1–2	6 (2.5%)	50 (20.7%)		**0.594**
Grade 3–4	5 (2.1%)	4 (1.7%)		**0.031**

Abbreviations: CC = concurrent chemotherapy, IMRT = intensity-modulated radiotherapy

## DISCUSSION

The most appealing finding of this large scale propensity score matched study is that the addition of cisplatin-based concurrent chemotherapy to IMRT did not lower the risk of death, locoregional relapse or distant metastasis in stage T1N1M0, T2N0-1M0 and T3N0M0 NPC.

Concurrent chemotherapy is recommended as the standard additional treatment to radiotherapy for NPC patients except for those with stage T1N0M0 disease by the National Comprehensive Cancer Network (NCCN). As locoregionally advanced NPC has high risk of locoregional relapse and distant metastasis, the addition of concurrent chemotherapy to radiotherapy can improve survival via the eradication of micrometastasis and enhancement of radiosensitivity [7–13]. It seems reasonable for the recommendation of radiotherapy plus concurrent chemotherapy to stage II NPC patients by the NCCN, according to the result of a recent study by Chen et al [4]. However, all the included patients in that study underwent conventional radiotherapy using a two-dimensional technique [4], which was inferior to IMRT in local tumor control, especially in the early T-stage patients [5]. Thus we considered that the survival benefit from concurrent chemotherapy in the 2DCRT era was possibly replaced by the survival advantage of IMRT. For example, the 4-years OS, DMFS and LRFS rates for IMRT alone in the present study (97.4%, 96.5% and 93.8%, respectively) were quite similar to those for 2DCRT plus concurrent chemotherapy in the study by Chen et al (97.4%, 97.3% and 95.7%, respectively) [4]. Secondly, stage II in that study [4] was defined by the Chinese 1992 staging system, and 31 patients were actually staged N2 according to the 2010 UICC/AJCC staging system. The known significant survival benefit from concurrent chemotherapy in the subgroup of 31 patients with stage N2 [7–13] might falsely cause the survival benefit for all the included patients in that study [4]. Additionally, previous retrospective comparison of 2DCRT alone with 2DCRT plus concurrent chemotherapy in 392 patients with T2N1M0 NPC (the 2002 UICC/AJCC staging system) showed no significant differences in OS or disease-free survival despite the improvement of LRFS [14]. Thus it is less likely to achieve survival gain from concurrent chemotherapy when IMRT has significantly improved the locoregional control.

In the IMRT era, Tham et al [15] attempted to justify the omission of chemotherapy in 107 patients with stage IIb (the 1997 AJCC staging system). The comparable survival rates between patients with and without any chemotherapy strategies (including abbreviated neoadjuvant chemotherapy, concurrent chemotherapy and adjuvant chemotherapy) indicated that IMRT alone might be sufficient treatment for this particular subgroup of patients. The insignificant differences in any survival endpoints between patients with and without concurrent chemotherapy also supported the plausibility of IMRT alone, albeit that only eight (7.5%) patients in that study received concurrent chemotherapy. Conversely, a most recent study by Kang et al [16] observed benefit from concurrent chemotherapy to stage II (the 2002 UICC/AJCC staging system) NPC in locoregional control and progression-free survival, but not DMFS or OS. Of note, among the 41 patients without concurrent chemotherapy (seven patients received induction chemotherapy and one patients received adjuvant chemotherapy), 37 (90.2%) patients underwent three-dimensional conformal radiotherapy or IMRT, but they only achieved a 5-years LRFS of 66.6%, which was quite lower than the reported 5-years LRFS rate of 94.2% resulting from IMRT alone in the study by Su et al [17], and even similar to the 5-years LRFS of locoregionally advanced NPC treated with 2DCRT alone [8, 12].

So it was not absurd regarding the insignificant survival differences between IMRT alone and IMRT plus concurrent chemotherapy for low risk NPC in our study. Certainly, the average dose of cisplatin delivered in concurrent chemotherapy was lower than 200 mg/m^2^. Thus further prospective studies are warranted to confirm whether this should be responsible for the observed insignificant effect, as retrospective studies suggested that cumulative dose of cisplatin over 200 mg/m^2^ resulted in better OS in stage IIb and III (the 2002 UICC/AJCC staging system) patients who received 2DCRT or IMRT [18]. Possibly, concurrent chemotherapy with other more efficacious regimen might improve the survival. For instance, the induction regimen of taxanes (docetaxel or paclitaxel) plus cisplatin and fluorouracil (PF) was superior to PF alone in head and neck cancer [19–22] and neoadjuvant docetaxel and cisplatin significantly improved OS of advanced NPC when comparing to chemoradiotherapy alone [23]. Thus taxanes-based concurrent chemotherapy might be a potentially effective alternative [24, 25]. Additionally, concurrent chemotherapy with small molecular targeted drugs such as endostar (e.g., NCT02237924), nimotuzumab (e.g., NCT01074021) and bevacizumab [26] deserved further investigation in early stage patients. Despite the fact that these patients staged with T1N1M0, T2N0-1M0 or T3N0M0 usually have low risk of relapse and distant metastasis on the whole, selecting patients using molecular biomarkers [e.g., deoxyribonucleic acid (DNA) copy number of the Epstein-Barr virus] might be a valid approach to better survival.

Obviously, the treatment outcomes from the current study were higher than those from RTOG 0225 [27] and MSKCC [28] studies, but comparable to those from the similar early stage NPC study by Su et al [17]. The differences in tumor stage most possibly resulted in the big gap of survival between these studies. Specifically, 58.9% and 77% of patients included in RTOG 0225 and MSKCC studies were staged III and IV, respectively. These locoregionally advanced disease undoubtedly had low survival rate. Inversely, stage II patients had a 5-year OS rate of 85.8% and 5-year PFS rate of 77.8% when receiving 2DCRT without chemotherapy [4], and even achieved a 5-year disease specific survival rate of 97.3% from IMRT alone [17]. Secondly, only 33.8% of patients in RTOG 0225 (32% in MSKCC study) were Asian, and over 40% (35% in MSKCC study) of patients were diagnosed with WHO I/II histology. The differences in ethnicity and histology may also contribute to the survival disparities. For example, the 3-year OS rate was 90% in a report from Hong Kong [29], which was much higher than the 2-year OS rate of 80.2% in RTOG 0225. Finally, the small number of patients in RTOG 0225 and MSKCC study possibly caused the skewed results as well.

The major strength of this study lies in the investigation of concurrent chemotherapy effect in low risk NPC in the IMRT era with the largest sample size using propensity score matching and multivariate analysis. This greatly addressed the limitations of divergent confounders and selection bias associated with the retrospective assessment of observational data [30]. Of course, the unobserved differences between the two arms cannot be balanced or adjusted. Although the presented data was derived from a single institution in endemic area with expertise in diagnosing and treating this disease, it did provide the most convincing evidence before the final report of any phase 3 randomized controlled trial. Since data on DNA copy number of the Epstein-Barr virus was missing in most of cases, VCA-IgA and EA-IgA were taken as the surrogate.

The major limitation is the missing data on acute non-hematological and late toxicities because of the retrospective design and the long time span between the first and the last included case. The recorded hematological toxicities might also be inaccurate due to the absence of strict and regular detection during treatment. But the additional toxicity from concurrent chemotherapy and similar toxicity from IMRT in the two arms were expected. Owing to the low sensitivity rate of chest radiography compared with chest computed tomography (CT), some patients might be delayed in detecting lung metastasis and have falsely high DMFS rate as a consequence. But the intrinsic differences in DMFS might scarcely change, as the chance of delay was equal to patients in both arms. Another limitation caused by the retrospective design was the heterogeneity of chemotherapy regimens and doses, albeit we restricted to patients with cisplatin-based concurrent chemotherapy in the propensity-matched cohort. Yet this phenomenon was the exact representation of the clinical reality out of randomized controlled trials. Additionally, it was possible that patients in the IMRT alone arm had smaller tumor volume, for the absence of matching this characteristic because of the unavailable data in many cases. But importantly, prior study [31] indicated that the pretreatment tumor volume had limited prognostic value in early stage NPC compared with the usual T-stage and N-stage. Even though great tumor volume showed association with greater risk of local failure, this might be possibly compromised by the improved local control from IMRT [5]. Further prospective studies are warranted.

In conclusion, this propensity-matched study indicated no significant survival benefit from adding cisplatin-based concurrent chemotherapy to IMRT for low risk NPC with stage T1N1M0, T2N0-1M0 or T3N0M0. Further confirmation by prospectively randomized controlled trial is ongoing.

## MATERIALS AND METHODS

### Patients

Between March 2003 and February 2013, 661 biopsy-proven, non-metastatic and treatment-naïve NPC patients who were at the age of 20 or above were entered into this study. All patients had complete pretreatment evaluation including patient history, physical examination, hematology and biochemistry profiles, fiberoptic nasopharyngoscopy with biopsy, magnetic resonance imaging (MRI) of the nasopharynx and neck, chest radiography or CT, abdominal sonography or CT, and Technetium-99m-methylene diphosphonate (Tc-99-MDP) whole-body bone scan or CT/MRI of bones. All the 661 patients were restaged with T1N1M0, T2N0-1M0 or T3N0M0 in accordance with the 2010 UICC/AJCC staging system for NPC.

### Treatment

All patients were treated by definitive IMRT with or without concurrent chemotherapy. The cumulative radiation doses were 66 Gy or greater to the primary tumor, 60–66 Gy to the involved cervical lymph nodes and 50 Gy or greater to potential sites of local infiltration and bilateral cervical lymphatics in 30–33 fractions. Further details of the radiation technique have been described previously [32]. Concurrent chemotherapy mainly consisted of 80–100 mg/m^2^ cisplatin- or nedaplatin-based regimen given every three weeks for two to three cycles, or 30–40 mg/m^2^ cisplatin- or nedaplatin-based regimen or 20–30 mg/m^2^ docetaxel-based regimen given weekly for up to seven cycles.

### Follow-up

Patients were examined every 3–6 months during the first 3 years, and every 6–12 months thereafter until death. During this period, patients were assessed by history and physical examination and a series of conventional examination equipment (e.g., fiberoptic nasopharyngoscopy, MRI of the nasopharynx and neck, and distant metastastic work-up if indicated.) at each follow-up visit, to detect the possible relapse or distant metastasis. Local relapses were confirmed by biopsy, MRI scan, or both. Regional relapses were diagnosed by clinical examination and MRI scan of the neck and, in doubtful cases, by fine needle aspiration of the lymph nodes. Distant metastases were diagnosed by clinical symptoms, physical examinations, and imaging methods including chest radiography or CT, Tc-99-MDP whole-body bones scan or CT/MRI of bones, and abdominal sonography or CT. Patients without recent examination tests in the medical records were followed up by telephone call.

### Statistical analysis

To reduce the interference of treatment heterogeneity, only 306 patients treated with IMRT plus cisplatin-based concurrent chemotherapy were selected to match those treated with IMRT alone using propensity score matching method. This method creates similar case (IMRT alone) and control (IMRT plus cisplatin-based concurrent chemotherapy) arms with balanced but not equal characteristics, and reduces possible biases to a minimum in a retrospective analysis [30]. Propensity scores were computed by logistic regression for each patient based on the following covariates, age, sex, histology (WHO II, differentiated non-keratinising carcinoma; WHO III, undifferentiated non-keratinising carcinoma [33]), titers of VCA-IgA and EA-IgA, T-stage, N-stage and clinical stage. Patients were then matched without replacement at the ratio of 1:1 on those scores, rather than the individual covariates. Covariates balance between the two sets were examined by *t* test (continuous variable), χ^2^ test (categorical variable) and standardized difference [34] for the original unmatched and propensity-matched cohorts.

OS (time from treatment to death from any cause), DMFS (time from treatment to the first distant metastasis) and LRFS (time from treatment to the first locoregional relapse) were estimated with the Kaplan–Meier method [35] and compared with log-rank test. Adjusted hazard ratios with 95% confidence intervals (with IMRT plus cisplatin-based concurrent chemotherapy as reference) were calculated using Cox proportional hazards model [36]. In the propensity-matched cohort, survival curves were compared using stratified log-rank test by matched pairs, and hazard ratios were estimated using Cox proportional hazards model with a robust variance estimator to account for the clustering within matched pairs [37]. Toxicities in both arms were compared with χ^2^ test and standardized difference [34].

All statistical analyses were performed using IBM SPSS Statistics version 22.0 and Stata version 12.0. Two-sided *P* values < 0.05 and standardized difference > 0.10 [38] were considered to be significantly different.

## References

[1] Ferlay J, Bray F, Pisani P, Parkin DM (2004). Cancer Incidence, Mortality and Prevalence Worldwide.

[2] Cao SM, Simons MJ, Qian CN (2011). The prevalence and prevention of nasopharyngeal carcinoma in China. Chin J Cancer.

[3] Chang ET, Adami HO (2006). The enigmatic epidemiology of nasopharyngeal carcinoma. Cancer Epidemiol Biomarkers Prev.

[4] Chen QY, Wen YF, Guo L, Liu H, Huang PY, Mo HY, Li NW, Xiang YQ, Luo DH, Qiu F, Sun R, Deng MQ, Chen MY (2011). Concurrent chemoradiotherapy vs radiotherapy alone in stage II nasopharyngeal carcinoma: phase III randomized trial. J Natl Cancer Inst.

[5] Lai SZ, Li WF, Chen L, Luo W, Chen YY, Liu LZ, Sun Y, Lin AH, Liu MZ, Ma J (2011). How does intensity-modulated radiotherapy versus conventional two-dimensional radiotherapy influence the treatment results in nasopharyngeal carcinoma patients?. Int J Radiat Oncol Biol Phys.

[6] Sturmer T, Joshi M, Glynn RJ, Avorn J, Rothman KJ, Schneeweiss S (2006). A review of the application of propensity score methods yielded increasing use, advantages in specific settings, but not substantially different estimates compared with conventional multivariable methods. J Clin Epidemiol.

[7] Al-Sarraf M, LeBlanc M, Giri PG, Fu KK, Cooper J, Vuong T, Forastiere AA, Adams G, Sakr WA, Schuller DE, Ensley JF (1998). Chemoradiotherapy versus radiotherapy in patients with advanced nasopharyngeal cancer: phase III randomized Intergroup study 0099. J Clin Oncol.

[8] Lin JC, Jan JS, Hsu CY, Liang WM, Jiang RS, Wang WY (2003). Phase III study of concurrent chemoradiotherapy versus radiotherapy alone for advanced nasopharyngeal carcinoma: positive effect on overall and progression-free survival. J Clin Oncol.

[9] Kwong DL, Sham JS, Au GK, Chua DT, Kwong PW, Cheng AC, Wu PM, Law MW, Kwok CC, Yau CC, Wan KY, Chan RT, Choy DD (2004). Concurrent and adjuvant chemotherapy for nasopharyngeal carcinoma: a factorial study. J Clin Oncol.

[10] Chan AT, Teo PM, Ngan RK, Leung TW, Lau WH, Zee B, Leung SF, Cheung FY, Yeo W, Yiu HH, Yu KH, Chiu KW, Chan DT (2002). Concurrent chemotherapy-radiotherapy compared with radiotherapy alone in locoregionally advanced nasopharyngeal carcinoma: progression-free survival analysis of a phase III randomized trial. J Clin Oncol.

[11] Wee J, Tan EH, Tai BC, Wong HB, Leong SS, Tan T, Chua ET, Yang E, Lee KM, Fong KW, Tan HS, Lee KS, Loong S (2005). Randomized trial of radiotherapy versus concurrent chemoradiotherapy followed by adjuvant chemotherapy in patients with American Joint Committee on Cancer/International Union against cancer stage III and IV nasopharyngeal cancer of the endemic variety. J Clin Oncol.

[12] Lee AW, Lau WH, Tung SY, Chua DT, Chappell R, Xu L, Siu L, Sze WM, Leung TW, Sham JS, Ngan RK, Law SC, Yau TK (2005). Preliminary results of a randomized study on therapeutic gain by concurrent chemotherapy for regionally-advanced nasopharyngeal carcinoma: NPC-9901 Trial by the Hong Kong Nasopharyngeal Cancer Study Group. J Clin Oncol.

[13] Blanchard P, Lee A, Marguet S, Leclercq J, Ng WT, Ma J, Chan AT, Huang PY, Benhamou E, Zhu G, Chua DT, Chen Y, Mai HQ (2015). Chemotherapy and radiotherapy in nasopharyngeal carcinoma: an update of the MAC-NPC meta-analysis. Lancet Oncol.

[14] Xu T, Hu C, Wang X, Shen C (2011). Role of chemoradiotherapy in intermediate prognosis nasopharyngeal carcinoma. Oral Oncol.

[15] Tham IW, Lin S, Pan J, Han L, Lu JJ, Wee J (2010). Intensity-modulated radiation therapy without concurrent chemotherapy for stage IIb nasopharyngeal cancer. Am J Clin Oncol.

[16] Kang MK, Oh D, Cho KH, Moon SH, Wu HG, Heo DS, Ahn YC, Park K, Park HJ, Park JS, Keum KC, Cha J, Kim JW (2015). Role of Chemotherapy in Stage II Nasopharyngeal Carcinoma Treated with Curative Radiotherapy. Cancer Res Treat.

[17] Su SF, Han F, Zhao C, Chen CY, Xiao WW, Li JX, Lu TX (2012). Long-term outcomes of early-stage nasopharyngeal carcinoma patients treated with intensity-modulated radiotherapy alone. Int J Radiat Oncol Biol Phys.

[18] Loong HH, Ma BB, Leung SF, Mo F, Hui EP, Kam MK, Chan SL, Yu BK, Chan AT (2012). Prognostic significance of the total dose of cisplatin administered during concurrent chemoradiotherapy in patients with locoregionally advanced nasopharyngeal carcinoma. Radiother Oncol.

[19] Posner MR, Hershock DM, Blajman CR, Mickiewicz E, Winquist E, Gorbounova V, Tjulandin S, Shin DM, Cullen K, Ervin TJ, Murphy BA, Raez LE, Cohen RB (2007). Cisplatin and fluorouracil alone or with docetaxel in head and neck cancer. N Engl J Med.

[20] Vermorken JB, Remenar E, van Herpen C, Gorlia T, Mesia R, Degardin M, Stewart JS, Jelic S, Betka J, Preiss JH, van den Weyngaert D, Awada A, Cupissol D (2007). Cisplatin, fluorouracil, and docetaxel in unresectable head and neck cancer. N Engl J Med.

[21] Lorch JH, Goloubeva O, Haddad RI, Cullen K, Sarlis N, Tishler R, Tan M, Fasciano J, Sammartino DE, Posner MR (2011). Induction chemotherapy with cisplatin and fluorouracil alone or in combination with docetaxel in locally advanced squamous-cell cancer of the head and neck: long-term results of the TAX 324 randomised phase 3 trial. Lancet Oncol.

[22] Hitt R, Lopez-Pousa A, Martinez-Trufero J, Escrig V, Carles J, Rizo A, Isla D, Vega ME, Marti JL, Lobo F, Pastor P, Valenti V, Belon J (2005). Phase III study comparing cisplatin plus fluorouracil to paclitaxel, cisplatin, and fluorouracil induction chemotherapy followed by chemoradiotherapy in locally advanced head and neck cancer. J Clin Oncol.

[23] Hui EP, Ma BB, Leung SF, King AD, Mo F, Kam MK, Yu BK, Chiu SK, Kwan WH, Ho R, Chan I, Ahuja AT, Zee BC (2009). Randomized phase II trial of concurrent cisplatin-radiotherapy with or without neoadjuvant docetaxel and cisplatin in advanced nasopharyngeal carcinoma. J Clin Oncol.

[24] Hu W, Ding W, Yang H, Shao M, Wang B, Wang J, Wu S, Wu S, Jin L, Ma CC (2009). Weekly paclitaxel with concurrent radiotherapy followed by adjuvant chemotherapy in locally advanced nasopharyngeal carcinoma. Radiother Oncol.

[25] Wei WH, Cai XY, Xu T, Zhang GY, Wu YF, Feng WN, Lin L, Deng YM, Lu QX, Huang ZL (2012). Concurrent weekly docetaxel chemotherapy in combination with radiotherapy for stage III and IVA-B nasopharyngeal carcinoma. Asian Pac J Cancer Prev.

[26] Lee NY, Zhang Q, Pfister DG, Kim J, Garden AS, Mechalakos J, Hu K, Le QT, Colevas AD, Glisson BS, Chan AT, Ang KK (2012). Addition of bevacizumab to standard chemoradiation for locoregionally advanced nasopharyngeal carcinoma (RTOG 0615): a phase 2 multi-institutional trial. Lancet Oncol.

[27] Lee N, Harris J, Garden AS, Straube W, Glisson B, Xia P, Bosch W, Morrison WH, Quivey J, Thorstad W, Jones C, Ang KK (2009). Intensity-modulated radiation therapy with or without chemotherapy for nasopharyngeal carcinoma: radiation therapy oncology group phase II trial 0225. J Clin Oncol.

[28] Wolden SL, Chen WC, Pfister DG, Kraus DH, Berry SL, Zelefsky MJ (2006). Intensity-modulated radiation therapy (IMRT) for nasopharynx cancer: update of the Memorial Sloan-Kettering experience. Int J Radiat Oncol Biol Phys.

[29] Kwong DL, Pow EH, Sham JS, McMillan AS, Leung LH, Leung WK, Chua DT, Cheng AC, Wu PM, Au GK (2004). Intensity-modulated radiotherapy for early-stage nasopharyngeal carcinoma: a prospective study on disease control and preservation of salivary function. Cancer.

[30] D'Agostino RB (1998). Propensity score methods for bias reduction in the comparison of a treatment to a non-randomized control group. Stat Med.

[31] Chua DT, Sham JS, Leung LH, Tai KS, Au GK (2004). Tumor volume is not an independent prognostic factor in early-stage nasopharyngeal carcinoma treated by radiotherapy alone. Int J Radiat Oncol Biol Phys.

[32] Sun X, Su S, Chen C, Han F, Zhao C, Xiao W, Deng X, Huang S, Lin C, Lu T (2014). Long-term outcomes of intensity-modulated radiotherapy for 868 patients with nasopharyngeal carcinoma: An analysis of survival and treatment toxicities. Radiother Oncol.

[33] Shanmugaratnam K, Sobin LH, Shanmugaratnam K, Sobin LH (1991). Histological typing of tumors of upper respiratory tract and ear. International histological classification of tumours.

[34] Austin PC (2009). Balance diagnostics for comparing the distribution of baseline covariates between treatment groups in propensity-score matched samples. Stat Med.

[35] Kaplan EL, Meier P (1958). Nonparametric estimation from incomplete observation. J Am Stat Assoc.

[36] Cox DR (1972). Regression models and life tables. J R Stat Soc B.

[37] Austin PC (2014). The use of propensity score methods with survival or time-to-event outcomes: reporting measures of effect similar to those used in randomized experiments. Stat Med.

[38] Normand ST, Landrum MB, Guadagnoli E, Ayanian JZ, Ryan TJ, Cleary PD, McNeil BJ (2001). Validating recommendations for coronary angiography following acute myocardial infarction in the elderly: a matched analysis using propensity scores. J Clin Epidemiol.

